# Maternal control of triploid seed development by the *TRANSPARENT TESTA 8* (*TT8*) transcription factor in *Arabidopsis thaliana*

**DOI:** 10.1038/s41598-023-28252-5

**Published:** 2023-01-24

**Authors:** Cecilia Zumajo-Cardona, Manuel Aguirre, Rosa Castillo-Bravo, Chiara Mizzotti, Maurizio Di Marzo, Camilla Banfi, Marta A. Mendes, Charles Spillane, Lucia Colombo, Ignacio Ezquer

**Affiliations:** 1 Dipartimento Di BioScienze, Università Degli Studi Di Milano, Via Celoria 26, 20133 Milano, Italy; 2 Genetics and Biotechnology Laboratory, Plant and AgriBioscience Research Centre (PABC), Ryan Institute, National University of Ireland Galway, University Road, Galway, H91 REW4 Ireland; 3 Present Address: Translational Plant & Microbial Biology, Institute of Environmental Biology, Utrecht University, Padualaan 8, 3584CH Utrecht, The Netherlands

**Keywords:** Differentiation, Developmental biology, Experimental organisms, Model plants, Plant sciences, Plant development, Plant reproduction

## Abstract

The balance between parental genome dosage is critical to offspring development in both animals and plants. In some angiosperm species, despite the imbalance between maternally and paternally inherited chromosome sets, crosses between parental lines of different ploidy may result in viable offspring. However, many plant species, like *Arabidopsis thaliana*, present a post-zygotic reproductive barrier, known as triploid block which results in the inability of crosses between individuals of different ploidy to generate viable seeds but also, in defective development of the seed. Several paternal regulators have been proposed as active players in establishing the triploid block. Maternal regulators known to be involved in this process are some flavonoid biosynthetic (FB) genes, expressed in the innermost layer of the seed coat. Here we explore the role of selected flavonoid pathway genes in triploid block, including *TRANSPARENT TESTA 4 *(*TT4*)*, TRANSPARENT TESTA 7 *(*TT7*)*, SEEDSTICK *(*STK*)*, TRANSPARENT TESTA 16 *(*TT16*)*, TT8* and *TRANSPARENT TESTA 13 *(*TT13*). This approach allowed us to detect that *TT8*, a bHLH transcription factor, member of this FB pathway is required for the paternal genome dosage, as loss of function *tt8*, leads to complete rescue of the triploid block to seed development.

## Introduction

In angiosperms, seeds are genetically chimeric structures formed upon double fertilization. The maternally-derived diploid seed coat (differentiated from ovule integuments), encloses the embryo resulting from the fusion of an haploid sperm cell with the haploid egg cell (diploid tissue), while the endosperm derives from the fusion of the diploid central cell with another haploid sperm cell^1–3^. To ensure proper seed development, complex crosstalk signalling between different seed compartments is required^4^. In fact, the innermost layer of the seed coat, the endothelium, which is in direct contact with the endosperm, has been reported to play a key role in this process^5,6^, being also, the only cell layer of the seed where the synthesis of proanthocyanidins occurs^6,7^. The proper development of the seed, after fertilization, is a process tightly controlled by several regulators such as genes specific to each compartment of the seed, hormones, and genes with maternal or paternal imprint^2,4,8–10^. This implies that changes in the ploidy of the parental lines can lead to defects in seed development and in several cases to seed unviability^3^. In fact, the triploid endosperm has a genomic ratio of 2 m:1p between the maternal and paternal genomes and several studies have demonstrated that alterating the 2 m:1p genomic ratio of endosperm leads to dramatic consequences^11,12^, that can cause failure of the development of F1 seed^11,13,14^. This phenomenon is known as the triploid block, which is a post-zygotic reproductive barrier tightly linked to parental genome dosage effects in the F1 seeds arising from interploidy crosses^3^.

Although polyploidization plays a prominent role in plant evolution^15–18^ and despite the agronomic importance of interploidy crosses, the molecular mechanisms responsible for the successful formation of triploid seeds are not yet fully understood^19^. Evidence suggests that molecular mechanisms regulating the triploid block are likely related to parent-of-origin small RNA dosage and to histone modifications in the developing endosperm^20,21^. In *Arabidopsis thaliana* the response to parental genome imbalance, resulting from interploidy crosses, depends on the genetic background of the accessions used in the crosses. Whereas interploidy crosses within the Columbia ecotype (Col-0 or Col) genetic background result in a strong triploid block, interploidy crosses within the Landsberg erecta (L*er*) or C24 genetic backgrounds show only a weak or partial triploid block^22^.

In addition, maternal versus paternal genome dosage excess affects seed size^23–25^. Precocious endosperm cellularization and reduced F1 seed size are correlated with F1 seeds having maternal-excess genome dosage, which in some cases results in seed abortion^23,24^. Excess in the paternal genomic dosage, on the other hand, is associated with endosperm over-proliferation and cellularization failure, leading to larger F1 seeds, including collapsed and/or aborted seeds^25–27^.

Two main molecular mechanisms have been suggested to explain such effects in seed development as result of the unbalanced ratio of maternal/paternal genomic dosage in F1 offspring^6,28^. First, that the altered expression of imprinted genes in the endosperm would lead to cellularization defects, and possibly to seed abortion^11,19,29–32^, and the second mechanism, involves a maternal-zygotic crosstalk possibly involving flavonoid biosynthetic genes.

Endosperm development is also under the sporophytic maternal control of the seed integuments, alteration of such sporophytic maternal–zygotic crosstalk in interploidy crosses can interfere with developmental programs of the embryo, endosperm and seed coat, resulting in defective seed formation^6,33–37^.

**Figure 1:**
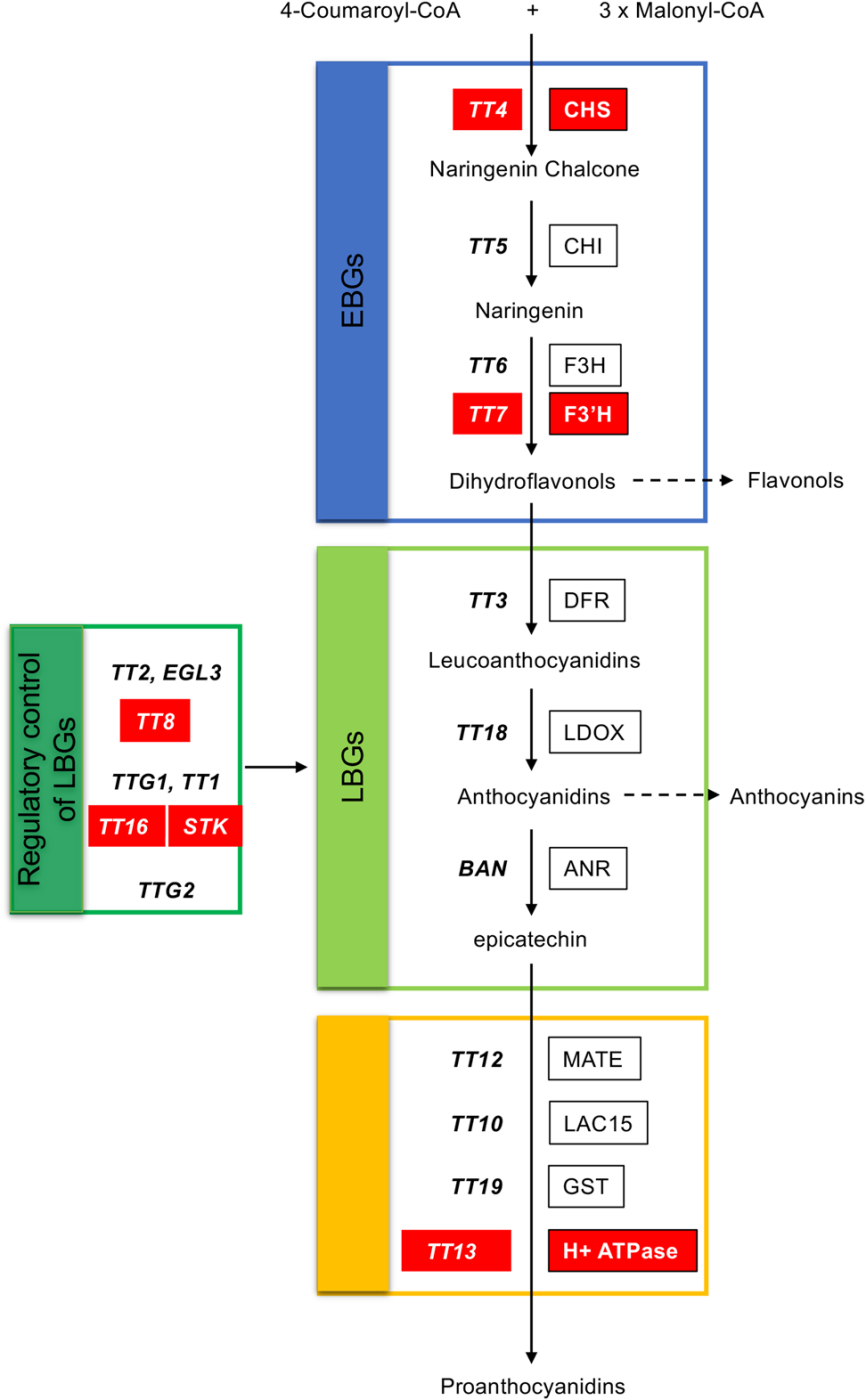
Schematic representation of the flavonoid biosynthetic pathway in *Arabidopsis thaliana* seeds. Genes encoding enzymes for each step are indicated as follows: (1) Early biosynthesis genes: *TRANSPARENT TESTA 4* (*TT4*), *TRANSPARENT TESTA 5* (*TT5*), *TRANSPARENT TESTA 6* (*TT6*) and *TRANSPARENT TESTA 7* (*TT7*), which are involved in the biosynthesis of PAs precursors and other classes of *Arabidopsis* flavonoids. (2) Later biosynthesis genes (LBG): *TRANSPARENT TESTA 3* (*TT3*), *TRANSPARENT TESTA 18* (*TT18*) and BANYULS/ANTHOCYANIDIN REDUCTASE (BAN/ANR). 3) *TRANSPARENT TESTA 12* (*TT12*, MATE transporter), *TT10* (laccase 15), *TT19* (Glutathione-S-transferase) and *TRANSPARENT TESTA 13* (*TT13*). The regulatory control of LBGs requires the action of a MBW transcriptional regulation complex (MYB-bHLH-WDR), formed by a specific R2R3-MYB (TT2), bHLH transcription factors (*EGL3, TT8*) and a WD repeat protein *TRANSPARENT TESTA GLABRA* 1 (*TTG1*). Other transcription factors belonging to different families such as C2H2 and C2HC zinc finger (*TT1/WIP1*), MADS-box (*ABS/TT16/AGL32, STK*) and WRKY (*TTG2/DSL1/WRKY44*) also participate in their regulation.

Here we focused on the maternal regulators and their possible role in seed development, flavonoids and in particular proanthocyanidins (PAs) which are synthesized in the endothelium, the innermost layer of the seed coat, of maternal origin, where they accumulate and give the characteristic brown colour of *Arabidopsis thaliana* seeds upon oxidation^38,39^. PAs accumulation provides many benefits to seeds such as protection against UV-light, microbes and herbivores, in addition to antioxidant and allelopathic activities^40^. It has been suggested also, that PAs are involved in seed development resulting from interploidy crosses^6^. Mutation of maternally-expressed genes, such as *TRANSPARENT TESTA GLABRA 2* (*TTG2*) and *TRANSPARENT TESTA 4 *(*TT4*), involved in PA biosynthesis, a branch of the flavonoid biosynthesis pathway (FBP), can partially suppress F1 seed lethality caused by paternal-excess interploidy crosses in *Arabidopsis thaliana*^6,22,41^. Although much progress has been made in identifying genetic modifiers of the triploid block, most of the advances to date have focused on the paternal elements responsible for (i) the high sensitivity of *Arabidopsis thaliana* to increased paternal genome dosage and, (ii) the triploid block^31,42,43^. Thus, to have a better understanding of the possible role of flavonoids in triploid block, here we explore the role of several genes in the flavonoid biosynthetic pathway (i.e., *TT4, TT7, STK, TT16, TT8* and *TT13*; Fig. 1) in balanced and unbalanced crosses, by analyzing maternal parents carrying loss-of-function mutations and crossing them with paternal excess genomic ratio dosage plants (4x). Our results indicate that mutants of most of these genes, partially rescue triploid block unlike *TT8* which completely rescues the triploid block, which occurs in response to increased paternal genome dosage in *Arabidopsis thaliana* Col-0 ecotype. Also suggesting, that the maternal contribution of *TT8* transcription factor plays a major role for post-zygotic barriers and that the flavonoid biosynthetic pathway is not entirely responsible for triploid block, besides that the function of *TT8* in triploid block could be independent of the flavonoid biosynthetic pathway. TT8 which belongs to the bHLH gene family and, together with members of the MYB, bHLH and WD40 gene families forms the MBW complex^44–46^, also appears to interact with Aux/IAA proteins (http://bar.utoronto.ca). Our results from the study of triploid block, this complex mechanism that is still poorly understood and for which additional studies are required, allow us to propose novel hypotheses about the role of endothelium specific transcription factors in the interplay between maternal, paternal and zygotic control in F1 polyploid seeds.

## Results

### Maternal-specific mutations in FBP genes rescue the post-zygotic triploid block to seed development

**Figure 2:**
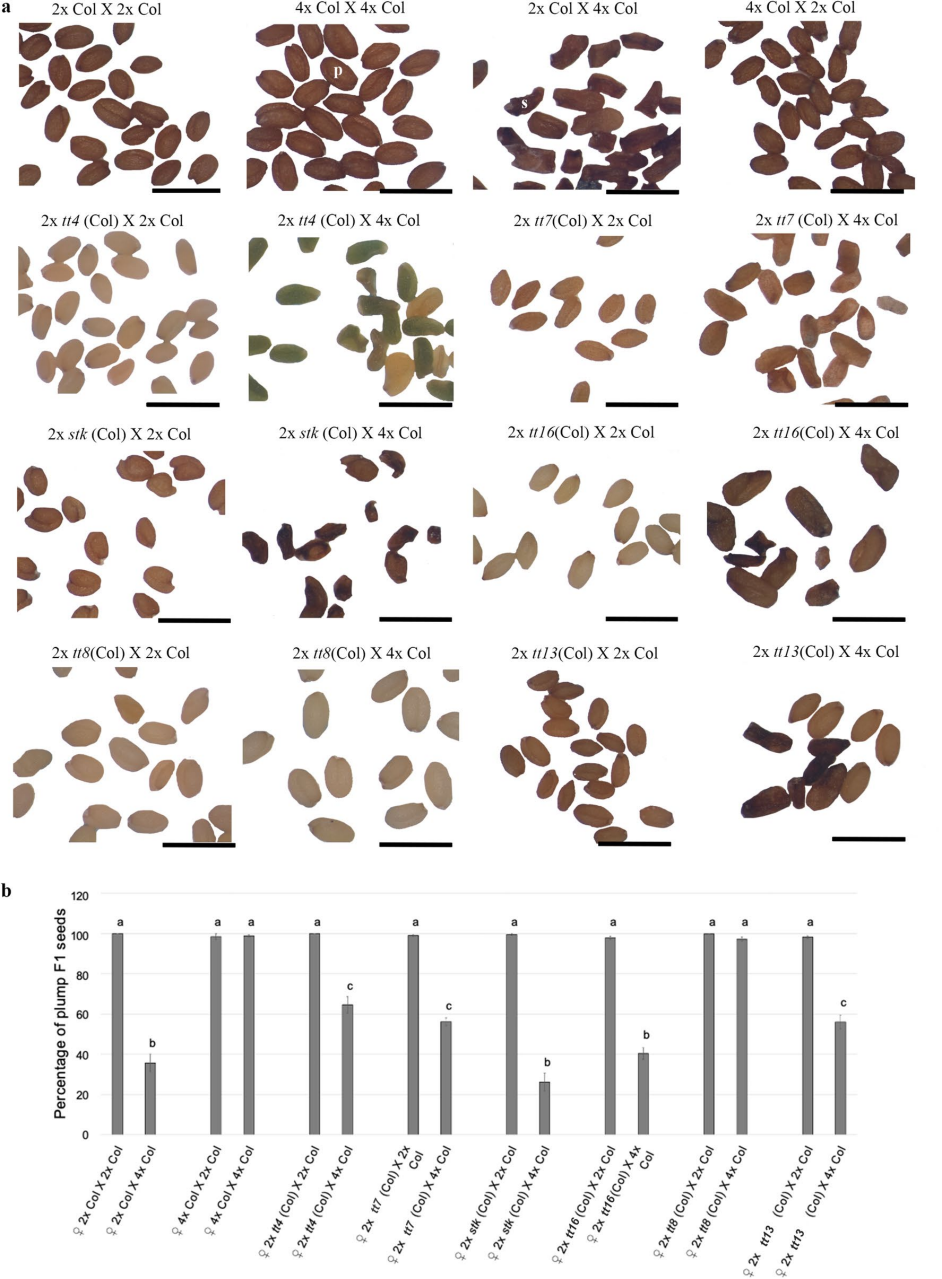
F1 seed shape of different FBP related mutants in interploidy crosses. (**a**) Mature F1 seeds obtained crossing FBP mutants in balanced and paternal-excess crosses. Wildtypes are shown on the first row. (**b**) Percentage of plump F1 seeds. Data are presented as means ± standard error. 200–300 F1 seeds were analyzed for each cross. Pooled data of three independent assays performed with 3 replicates for each measurement. One-way ANOVA followed by Tukey HSD test was used for analyzing significance. *tt4* is *tt4*-*11*; *tt7* is *tt7-2* ; *stk* is *stk-2*; *tt16* is *tt16-7*; *tt8* is *tt8-6*; *tt13* is *tt13-6.* Crosses marked with the same lowercase letter display no statistical difference. p: plump seeds; s: shriveled seeds, Col = Col-0. *Scale:* 1 mm (**a**).

To explore the possible role of flavonoids in the control of the post-zygotic triploid block in F1 seed development, we used loss-of-function mutants for genes involved in different steps of the flavonoid biosynthetic pathway, which also allows us to determine if there is any difference along this pathway in the control of triploid block. Thus, we tested mutants of several genes such as *TT4, FLAVANONE 3 HYDROXYLASE/ TRANSPARENT TESTA 7 *(*F3’H*/*TT7*)*, ARABIDOPSIS BSISTER/TRANSPARENT TESTA 16* (*ABS/TT16*), *SEEDSTICK* (*STK*), *TT8* and *AUTOINHIBITED H*( +)*-ATPASE ISOFORM 10/TRANSPARENT TESTA 13 *(*AHA10*/*TT13*)^47–49^ (Fig. 1)*. TT4,* acts in the first step of flavonoid biosynthesis, and it has already been reported that it partially rescues the post-zygote triploid block resulting from unbalanced crosses^22^. Homozygous loss-of-function mutants in each of the genes were used as maternal parents in interploidy (and euploidy) crosses with pollen from 2x (diploid) and 4x (tetraploid) plants^50^. The percentage of plump F1 seeds obtained in interploidy crosses of the 2 × mutants X 4 × Col were compared to the percentage of plump F1 seeds produced by 2 × Col X 2 × Col, 4 × Col X 4 × Col euploidy controls, and also in 2 × Col X 4 × Col (paternal-excess control) and 4 × Col X 2 × Col (maternal-excess control) (Fig. 2). The loss of function for both *TT4* and *TT7* mutants displayed a statistically significant increase in the number of F1 plump seeds (64% and 56%, respectively) when compared to the 35% of F1 plump seeds produced by the paternal-excess control crosses 2 × Col X 4 × Col (Fig. 2b). The plump F1 *tt4* seed percentage was equivalent to what was previously reported for *ttg2* (64%), which works upstream of *TT4*^22^ (Fig. 2b). In addition, the disruption of *TT13*, partially rescued the percentage of plump F1 seed phenotype (56% of plump seeds) (Fig. 2b).

Compared to the 2 × Col X 4 × Col cross, neither 2 × *stk* (Col) X 4 × Col nor 2 × *tt16* (Col) X 4 × Col, have shown any statistical difference from the paternal-excess control. In contrast, the 2 × *tt8* (Col) X 4 × Col cross rescued almost completely the percentage of plump F1 seeds (97%) (Fig. 2b).

To verify the role of *TT8* in maternal control of the triploid block, we analyzed four different *tt8* alleles namely *tt8-1*, *tt8-5*, *tt8-4*, *tt8-8* (Supplementary Fig. S1a), which allowed us to corroborate that all the *tt8* alleles fully recover the F1 plump seed phenotype from unbalanced crosses (Supplementary Fig. S1).

The bypass of the triploid block was further corroborated with germination tests on all alleles, showing that *tt8* mutations fully rescue seed viability after interploidy crosses (Supplementary Fig. S2). Indicating that the bypass of the triploid block may arise due to the lack of functional TT8 protein (Fig. 2).

Overall, these results indicate that loss-of-function mutations of sporophyte-expressed genes involved in the flavonoid biosynthetic pathway may partially, as occurs with *TT4, TT7, TT13* or completely, as occurs with *TT8*, bypass the triploid block triggered by an unbalanced zygotic genome dosage (Fig. 2).

**Figure 3:**
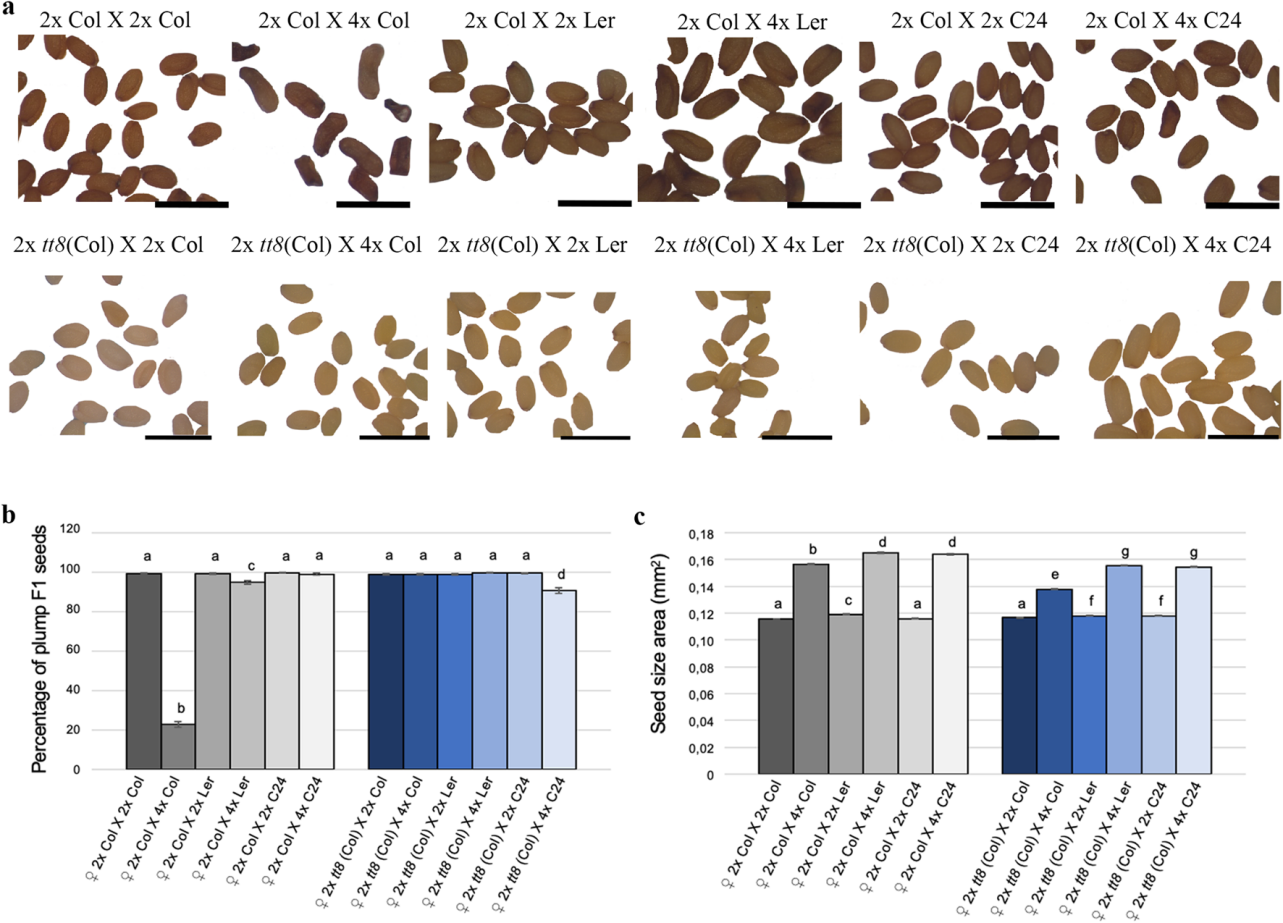
Interploidy crosses in wildtype and *tt8* using three accessions of *Arabidopsis thaliana* (Col, Ler and C24). (**a**) Mature F1 seeds obtained crossing diploid Col and *tt8* mutant with diploid or tetraploid pollen from three *Arabidopsis thaliana*. (**b**) Percentage of plump F1 seeds from interploidy crosses using different Arabidopsis accessions as pollen donors to pollinate diploid Col and *tt8* mutants. Data presented as means ± standard error. Around 200 F1 seeds were analyzed for each cross. Pooled data of three independent assays performed with 3 replicates for each measurement. (**c**) Seed size of F1 seeds resulting from interploidy crosses using three accessions of Arabidopsis pollen donors to pollinate diploid Col and 2 × *tt8* (Col) mutants. Data is presented as means ± standard error. Pooled data of three independent assays performed with 3 replicates for each treatment (200–300 seeds/each). *tt8* corresponds to *tt8-6.* One-way ANOVA followed by Tukey HSD test was used for analyzing significance. Crosses marked with the same lowercase letter display no statistical difference. *Scales:* 1 mm (a).

While the Col accession exhibited a strong triploid block to seed development in 2 × X 4 × crosses (paternal excess), the prevalence of shrivelled seeds generated was significant around 90%. Paternal-excess interploidy crosses with the accessions L*er* and C24, generated almost 100% plump seeds^51–53^ (Fig. 3a,b).

Paternal-excess interploidy crosses (2 × Col X 4 × Col) generated larger F1 seeds than seeds from 2 × Col X 2 × Col crosses^25^, mainly due to over-proliferation and defective cellularization of the endosperm, and abnormal development of the seed coat^6,24,54^. Consistent with previous studies^25^, we found an increase of approx. 34% of F1 seed size derived from 2 × Col X 4 × Col crosses, compared to F1 seeds derived from 2 × Col X 2 × Col crosses (here defined as the control) (Fig. 3b). All *tt8* alleles tested displayed a similar increase in F1 seed size and this was slightly lower than the control (Fig. 3c; Supplementary Fig. S1d).

The resulting F1 seeds from crosses with 4 × L*er* and 4 × C24, displayed a significant increase in seed size which was slightly lower when we pollinated 2 × *tt8* (Col) plants compared to the 2 × Col plants (Fig. 3c).

Eventhough maternal excess in wildtype *Arabidopsis thaliana* does not exhibit a post-zygotic barrier (i.e., 4 × Col X 2 × Col; produces viable seeds)^37^, it is unclear whether loss-of-function *tt8* results in any seed defects. To find out if *TT8* impacts seed development in maternal-excess interploidy crosses, we generated colchicine-induced nulliplex autotetraploid (4x) mutant lines of *tt8* (Col) that were then crossed with pollen from 2 × Col and 4 × Col wildtype (WT) lines (Supplementary Fig. S3), *tt4* analyses were also included (Supplementary Fig. S3). It was observed that 4 × *tt8* (Col) X 2 × or 4 × *tt4* (Col) X 2 × maternal-excess interploidy crosses had no influence on F1 seed development (Supplementary Fig. S3a,b).

Regarding seed size, the maternal-excess triploid seeds from 4 × Col X 2 × Col crosses exhibited smaller F1 seed size compared^37^ to 4 × Col X 4 × Col seeds (Supplementary Fig. S3c). The 4 × *tt8*, and 4 × *tt4* mutations showed a similar trend in seed size as the control situation (4 × Col X 2 × Col), showing a 40% reduction in seed size compared to balanced situation (4 × Col X 4 × Col) (Supplementary Fig. S3c).

**Figure 4:**
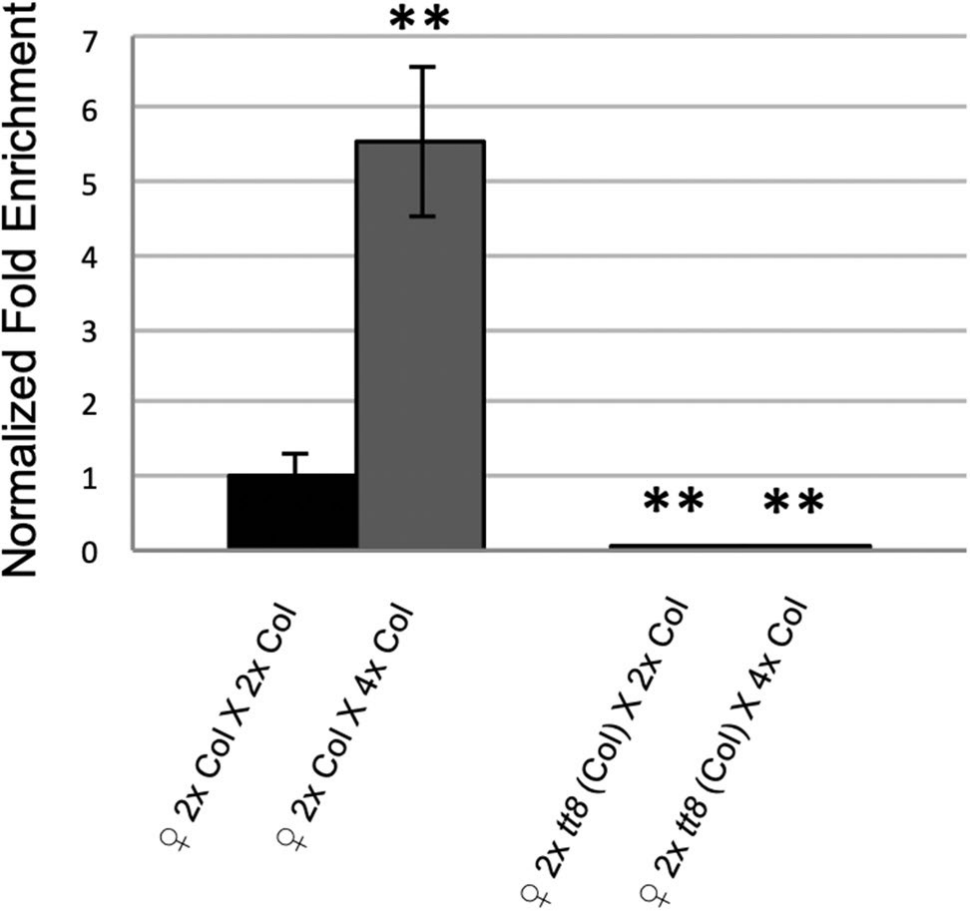
*TT8* mRNA expression levels in seeds at 72 h after pollination from balanced and paternal excess crosses. *tt8* corresponds to *tt8-6.* Data was normalized to *ACTIN*, with error bars indicating standard deviations based on four independent technical replicates. Statistical analysis was performed using Student’s t test (**p < 0.01). Three qPCR (biological replicates) were performed and generated similar results. Three independent biological replicates were performed.

*TT8* expression was strongly upregulated in paternal-excess crosses 2 × Col X 4 × Col, respect 2 × Col X 2 × Col (Fig. 4). *TT8* is reported to be specifically expressed in the endothelium after fertilization^54^. Indeed, in seeds obtained by crossing *tt8* with 2 × or 4 × pollen, *TT8* expression is not detectable, indicating that paternal *TT8* (wildtype) allele is not expressed in embryo or in the endosperm at the analyzed stages (Fig. 4). These results suggest that pollen ploidy might influence, at least in Col, the expression of seed coat specific genes such as *TT8*.

### TT8 controls the rate of endosperm proliferation to balance interploidy incompatibility

In *Arabidopsis thaliana*, endosperm proliferation begins after double fertilization, in coordination with the first mitotic divisions of the embryo^55^. Once endosperm development reaches its final volume, approximately 5 DAP the cellularization process begins^56^, simultaneously several genes repressing cell wall formation are silenced and active cytokinesis occurs in the syncytial endosperm^24,57^.

**Figure 5:**
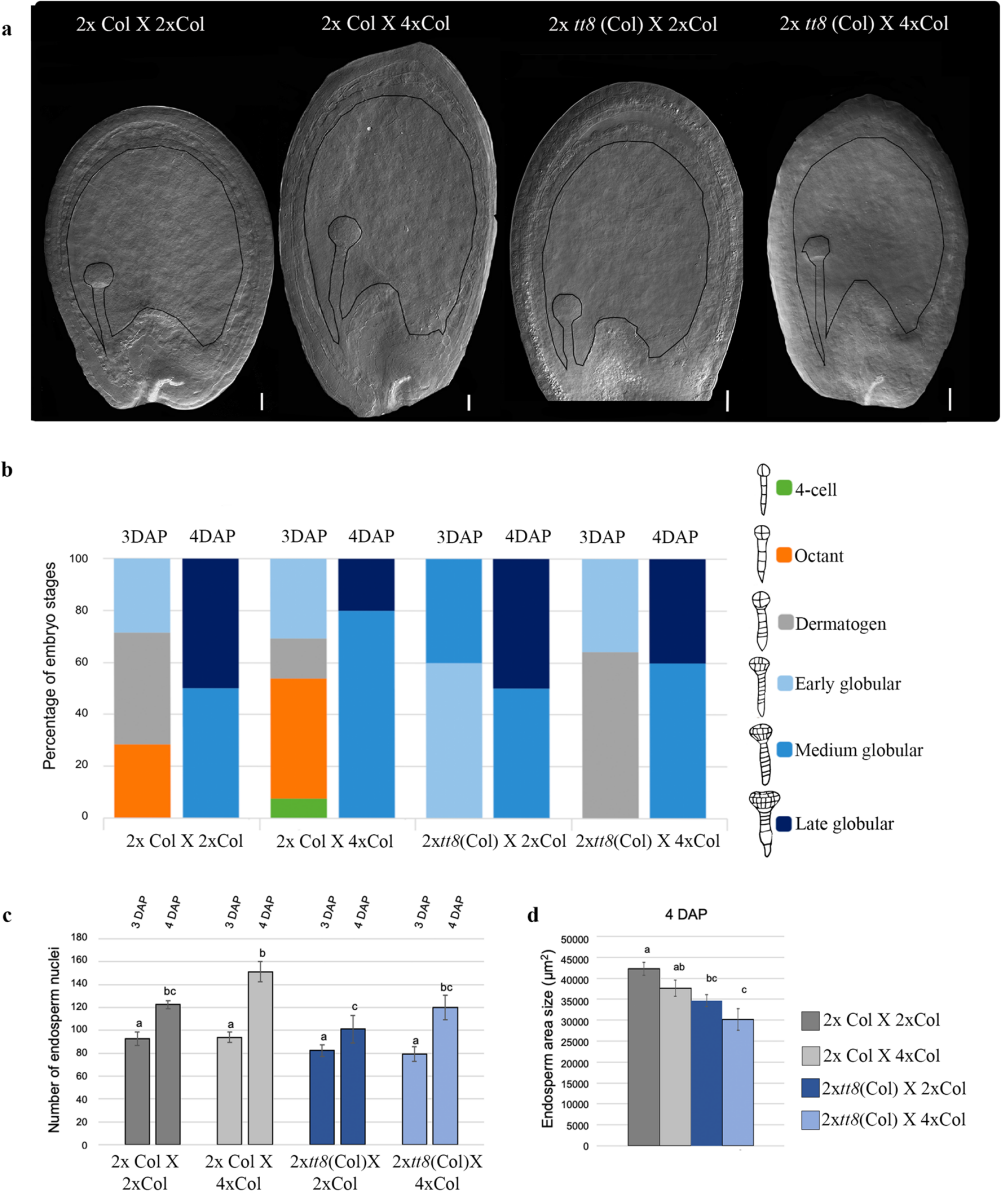
Timing of endosperm and embryo development following balanced and interploidy crosses. (**a**) Optical microscope image of cleared seeds from balanced and unbalanced crosses. (**b**) Percentage of embryo stages following balanced and interploidy crosses (percentages obtained from 20–25 seeds). (**c**) Endosperm proliferation following balanced and interploidy crosses at 3 and 4 DAP. Endosperm nuclei were counted in whole-mount seeds after clearing treatment. The mean number of endosperm nuclei ± standard error (n = 10 to 15) is shown for each cross. (**d**) Endosperm cavity area measurement following balanced and interploidy crosses at 4 DAP. Endosperm cavity areas were measured through whole-mount seeds after clearing treatment. The mean number of endosperm cavity area (µm2) ± standard error (n = 10 to 15) is shown for each cross. One-way ANOVA followed by Tukey HSD test was used for analyzing significance. *tt8* corresponds to *tt8-6.* Crosses marked with the same lowercase letter display no statistical difference. *Scale:* 20 μm (**a**).

To provide a more in-depth analysis of the role of TT8 in endosperm development, detailed measurement of endosperm proliferation, endosperm area, and characterization of embryo development was performed using a clearing protocol (Fig. 5a). Having found that embryo development appears relatively normal in paternal excess (2 × Col X 4 × Col) compared to control (2 × Col X 2 × Col). However, at 3 DAP, a slight delay in the development of the F1 seeds with paternal excess was observed, compared to the balanced cross (Fig. 5b). This delay was later compensated on the fourth day after pollination, showing embryos in the medium and late globular stage (Fig. 5b). The progeny of the F1 seed of the *tt8* mutant crossed with 4 × Col, also showed a minor delay compared to the progeny of the relative isogenic cross at 3 DAP, delay that appeared recovered at fourth day after pollination, then appearing similar to the control) (Fig. 5b).

Interestingly, at 4 DAP, our analyses indicated that paternal-excess crosses 2 × Col X 4 × Col showed an increment of approx. 25% in endosperm nuclei in Col wildtype, compared to 2 × Col X 2 × Col (Fig. 5c). The *tt8* mutant presented a lower number of endosperm nuclei when compared to F1 seed progeny from isogenic crosses, showing an approx. 20% reduction compared to 2 × Col X 2 × Col and from interploidy crosses 25% reduction compared to 2 × Col X 4 × Col (Fig. 5c). With this phenotype, the *tt8* mutant was found to display a reduced endosperm area in F1 seeds, both from balanced crosses 25% approx. reduction compared to 2 × Col X 2 × Col and unbalanced crosses 25% reduction compared to 2 × Col X 2 × Col (Fig. 5d).

Overall, we consider that the small endosperm area (Fig. 5d) may balance the developmental program leading to the maternal rescue observed in the *tt8* mutant interploidy crosses paternal-excess.

## Discussion

The triploid block resulting from interploidy crosses has been reported for over than five decades^58,59^. Recent studies, using the model species *Arabidopsis thaliana*, have mainly focused on the role of the fertilization product: endosperm and embryo in the establishment of the triploid block^29–31^, providing valuable information on the genetic regulatory factors that control it^29–31,59^. However, the potential role of maternally derived sporophytic tissues (seed coat) needs to be further explored.

It has been proposed that the flavonoid biosynthetic pathway, acting on the endothelium of the seed coat, may control triploid block. Genes such as TT4 and TTG2 that have been reported to impact the viability of seeds after unbalanced crosses. The results of this study show that the “lethal effect” observed in interploidy paternal excess crosses, in the Col ecotype can be bypassed by *TT8* loss-of-function mutations in all the *tt8* alleles assessed (Fig. 2, Supplementary Figs S1, S2). While the *tt8* mutant fully rescues the triploid block, our findings also show that loss-of-function mutations of other flavonoid biosynthetic pathway genes such as *TT4*, *TT7*, *TT13,* can only partially bypass the triploid block (Fig. 1, 2). Thus, triploid block rescue mediated by *tt8* knockout differs from the previously reported partial seed recovery in F1 seeds from paternal-excess crosses resulting from mutating FBP related genes^6,23^.

The assessment based on the percentages of plump seed phenotype, in both balanced and unbalanced F1 seeds shows that 98% of *tt8* knocked-out paternal excess seeds are plump, resembling wildtype balanced seed (2 × Col X 2 × Col; Fig. 3b), whereas only 65% of *tt4* mutant paternal excess seeds (2 × *tt4* Col x 4 × Col) are plump seeds. Seed size in *tt8* mutants is slightly reduced compared to wildtype seeds in both balanced and unbalanced F1 seeds (Fig. 3c) as has been reported for other seed coat specific genes such as *TTG2*^22^. These phenotypes are similar when using different *Arabidopsis thaliana* ecotypes (L*er* and C24) as pollen donors (Fig. 3).

Moreover, the high expression of *TT8* observed in unbalanced F1 seeds with paternal excess (2 × Col X 4 × Col), raises two probabilities: (1) that 2 × Col X 4 × Col seeds trigger the activation of TT8 transcription and, (2) that in these unbalanced crosses there has been a repression removed of TT8.

Embryo development is arrested in unbalanced seeds resulting from paternal excess crosses^37^ (Fig. 5) leading to unviability of seeds. In fact, *tt8* mutants show normal embryo development at the stages analysed here (Fig. 5). The effects of the triploid block are manifested in the endosperm since the seeds resulting from paternal excess do not show endosperm cellularization^13,28,60^. The cellularization process beginning 5 DAP while our observations, after having analysed seeds up to 4 DAP, allow us to determine that there is not premature cellularization detected. Our developmental characterization of triploid seeds corroborates a lack of cellularization in the balanced-ploidy crosses (2 × Col X 2 × Col and 4 × Col X 4 × Col) at 3 DAP^13,28,60^ (Figs. 5). In the *tt8* mutants resulting from balanced and imbalanced crosses it appears that endosperm development is not significantly affected (Fig. 5a,b). However, the reduced growth of the endosperm cavity in *tt8* mutants (Fig. 5c), translated into a reduction in the number of nuclei in the endosperm (Fig. 5d) that could possibly lead to an anticipated cellularization in paternal excess crosses. Also, the F1 seeds of the *tt8* mutant crossed with 4 × Col presented a reduced delay in embryo development compared to the progeny of the relative control exposed to unbalanced crosses. Therefore, these traits could be considered as part of the mechanisms responsible to bypass the triploid block in seeds from crosses with paternal excess^13,60^. This leads us to support the hypothesis that seed development is tightly coordinated between the seed coat, the embryo and endosperm developmental programs during normal seed development^35^. Given that the final seed size of the 2 × *tt8* (Col) X 4xCol is bigger in relation to the wildtype 2 × Col X 2 × Col (Fig. 3c) whereas the endosperm cavity 3 DAP is smaller (Fig. 5d), suggests that 1) the bigger seed size may be due to the width of the seed coat, or 2) the endosperm enlarges at later stages of seed development; a more detailed morphological characterization of the *tt8* mutant phenotype, will uncover its precise role in each tissue of the seed.

It is important to note that genes, involved in FBP, play a role in seed size regulation^6^. In balanced ploidy crosses, it is common for loss-of-function mutations of some FBP-related genes to produce smaller seeds^35,38,61,63^. The effect on seed size observed in these mutants, is considered to be associated with the precocious onset of endosperm cellularization^64^. The timing of endosperm proliferation is critical for the size of the embryo sac, which in turn is critical for defining the final size of the seed^58,65^. From the seed size assessment for *tt4* and *tt8* in interploidy crosses with maternal-excess (4 × Col X 2 × Col), we found no major differences (Supplementary Fig. S3).

Focusing on the triploid block that occurs in the endosperm, this study highlights seed coat-specific genes that play an important role in triploid block as they affect the endosperm. Underlying our findings, a cross-talk between the different tissues of the seed is evidenced^2,35^.

- Our findings, together with previous studies, show that although there are genes, members of the flavonoid biosynthetic pathway, that partially rescue triploid block, the function of *TT8* is crucial by fully rescuing it, and suggest in further that *TT8* function on triploid block may be independent of that in flavonoid biosynthesis.
- According to the *Arabidopsis thaliana* interactions viewer 2.0 (http://bar.utoronto.ca; Supplementary Fig. S4), TT8 interacts not only with members of the MBW complex of the FBP^45,46,66^, but also with other genes that are strongly expressed in the seed coat, including the repressor IAA27/PAP2 (Supplementary Fig. S4) a canonical Aux/IAA^67^. This suggests that IAA27 might repress TT8. Protein–protein interaction essays, are still required to corroborate this hypothesis however, in an unbalanced F1 seed (2 × Col X 4 × Col), endosperm cellularization does not occur as result of high auxin levels in the endosperm^68^ whereas in the seed coat, *TT8* is highly expressed (Fig. 4), and it has been previously shown that after fertilization auxin is transported from the endosperm to the seed coat^2,4,60^. Therefore, in F1 2 × Col X 4 × Col F1 seeds, higher levels of auxin in the endosperm activate *TT8* transcription via ubiquitination and degradation of IAA27. Corroborating previous results that auxin is involved in the coordinated development of the different seed compartments^4,60,67^.
- The early members of the flavonoid biosynthetic pathway such as TT4, which encodes the structural protein chalcone synthase, are involved in auxin transport via PIN and PGPs^69–71^, further affecting TT8 expression, via auxin signaling, which could explain the partial rescue of triploid block observed in *tt4* mutants (Fig. 2). And suggesting that the role of these genes in triploid block is not due to their function in the FBP per se, but rather to their role in auxin flux and signaling.

Altogether, our findings provide a better understanding of the role of seed coat-specific genes in seed development and triploid block, shedding light on novel mechanisms, and on TT8, an important regulator for proper seed development.

## Materials and methods

### Plant material and growth conditions

For this study the following *Arabidopsis thaliana* accessions and lines were used: wildtype (2 × ecotype Columbia-0, Col-0, 2 × ecotype Ler (Landsberg erecta), 2 × ecotype C24), as well as (4 × ecotype Columbia-0, Col-0, 4 × ecotype Ler (Landsberg erecta), 4 × ecotype C24) (obtained from Spillane Lab, National University of Ireland Galway, Ireland), *tt16-7* (T-DNA SALK_077737), *tt13-6* (T-DNA GK-170A07^47^), *tt7-2* (T-DNA SALK_053394^72^; later called *tt7-5*^73^), *stk-2*^74^, *tt4-11* (T-DNA SALK_020583^75^ later called *tt4-13*^73^), *tt8* (reported in TAIR as *tt8-6*) (T-DNA GK-241D05; 2nd intron). Other *tt8* alleles (Supplementary Fig. S1) that were used were the following: *tt8-4* (T-DNA SALK_030966; 1st exon^76^), *tt8-5* (T-DNA SALK_048673; 3rd exon), *tt8-14* (T-DNA SALK_063334; 5th intron), *tt8-15* (T-DNA SALK_121609; 3’UTR; new report according to conventional nomenclature^45^). Alleles *tt8-7* to *tt8-13* were not used in this study^77^. Plants were germinated at 22 °C under short-day conditions (8 h light/16 h darkness) and transferred to long day conditions (16 h light/ 8 h darkness). The mutant lines were already corroborated homozygous mutant plants. Plants were cultivated and collected following the relevant institutional, national, and international guidelines and legislation, with all the permissions and licenses for collec tion of plants/seeds.

### Colchicine treatments

The, *tt4* and *tt8* tetraploid mutant lines in Col background were generated by applying 0.1% (w/v) colchicine solution as described in to two-week-old diploid seedlings. The newly generated tetraploids were self-pollinated for three generations by single seed descent and the ploidy level was validated by flow cytometry using a BD Accuri™ C6 Plus flow device following the manufacturer’s instructions.

### Expression analysis

Flower buds that were just about to open were emasculated. After 24 h, emasculated pistils were manually pollinated by depositing the pollen from the donor plant on the stigmatic papillae of the receptor pistils. RNA from 20 to 25 manually dissected siliques at 3 DAP (Day After Pollination) after being pollinated were collected in NucleoProtect RNA stabilization reagent (Macherey–Nagel). 3 biological replicates were collected and RNA was obtained using the NucleoSpin RNA, Mini kit for RNA purification (Macherey–Nagel) following the supplier's instructions. Total RNA was retro-transcribed using the iScriptTM gDNA Clear cDNA Synthesis kit (Bio-Rad) following the supplier’s instruction. cDNAs were used as templates in the qRT-PCR reactions containing the iQ SYBR Green Supermix (Bio-Rad). The qRT-PCR assay was conducted in triplicate on different biological replicates, with three technical replicates for each sample, and was performed in a Bio-Rad iCycler iQ Optical System (software version 3.0a). Relative transcript enrichment of genes of interest was calculated normalizing the amount of mRNA against control fragments (ACT). Gene expression analysis was performed with the 2ˆ − ∆∆Ct method using the specific tool at Bio-Rad CFX Maestro software v.4.0 (Bio-Rad). The primers used for this analysis are listed in Suplementary Table S1.

### Seed Size analysis

Seeds were photographed using a Leica MZ6 stereomicroscope, and seed images were measured using smartgrain software^78^. Measurements were statistically analyzed by one-way ANOVA with a post-hoc Tukey honestly significant difference (HSD) comparison test.

### Morphological analysis of interploidy crosses

Microscopic observations were performed using a Zeiss Axiophot D1 microscope (http://zeiss.com/) equipped with differential interface contrast (DIC) optics. Images were recorded with an Axiocam MRc5 camera (Zeiss) using the Axiovision program (version 4.1). In *A. thaliana* floral stems, the self-pollinated siliques in formation and the already opened flowers were removed. The flower buds that were just about to open were emasculated. With the help of tweezers and under a stereomicroscope, all the floral organs were removed, except for the pistil, which contained the ovary. Within a period of 24 h after emasculation a first pollination was performed by depositing the pollen from the donor plant in the stigma of the receptor pistils. Within 24 h after first pollination, a second one was performed in the same way with the purpose of increasing the possibilities of obtaining a successful fecundation. It was considered that the crosses were successful when a few days after pollinating, the pollinated pistils grew until maturity and the resulting siliques contained seeds. Endosperm analyses were performed as previously reported^22^, 20 seeds per genotype at 3 DAP and 4 DAP were cleared^75^ and immediately photographed using a microscope. Endosperm size was measured in ImageJ (https://imagej.nih.gov/ij/) using the ‘analyze particles’ function.

### Accession Number

Sequence data from this article can be found in the GenBank/EMBL data bases under the following accession numbers: TT13 = At1g17260; CHS/TT4 = At5g13930; TT7 = At5g07990; TT8/bHLH042 = At4g09820; ABS/TT16/AGL32 = At5g23260; STK = At4g09960.

## Supplementary Information

Supplementary Information.

## Data Availability

The authors declare that all data supporting the findings in this study are available within the paper, Supplementary information and Source data. All data are available upon request to the corresponding author.
