# Supplementary material for: Maternal control of triploid seed development by the TRANSPARENT TESTA 8 (TT8) transcription factor in Arabidopsis thaliana
Source: Sci Rep. 2023 Jan 24;13:1316. doi: 10.1038/s41598-023-28252-5 (PMC9873634; doi:10.1038/s41598-023-28252-5)
Supplement: Supplementary file 1 — Supplementary Information. [file 41598_2023_28252_MOESM1_ESM.pdf]

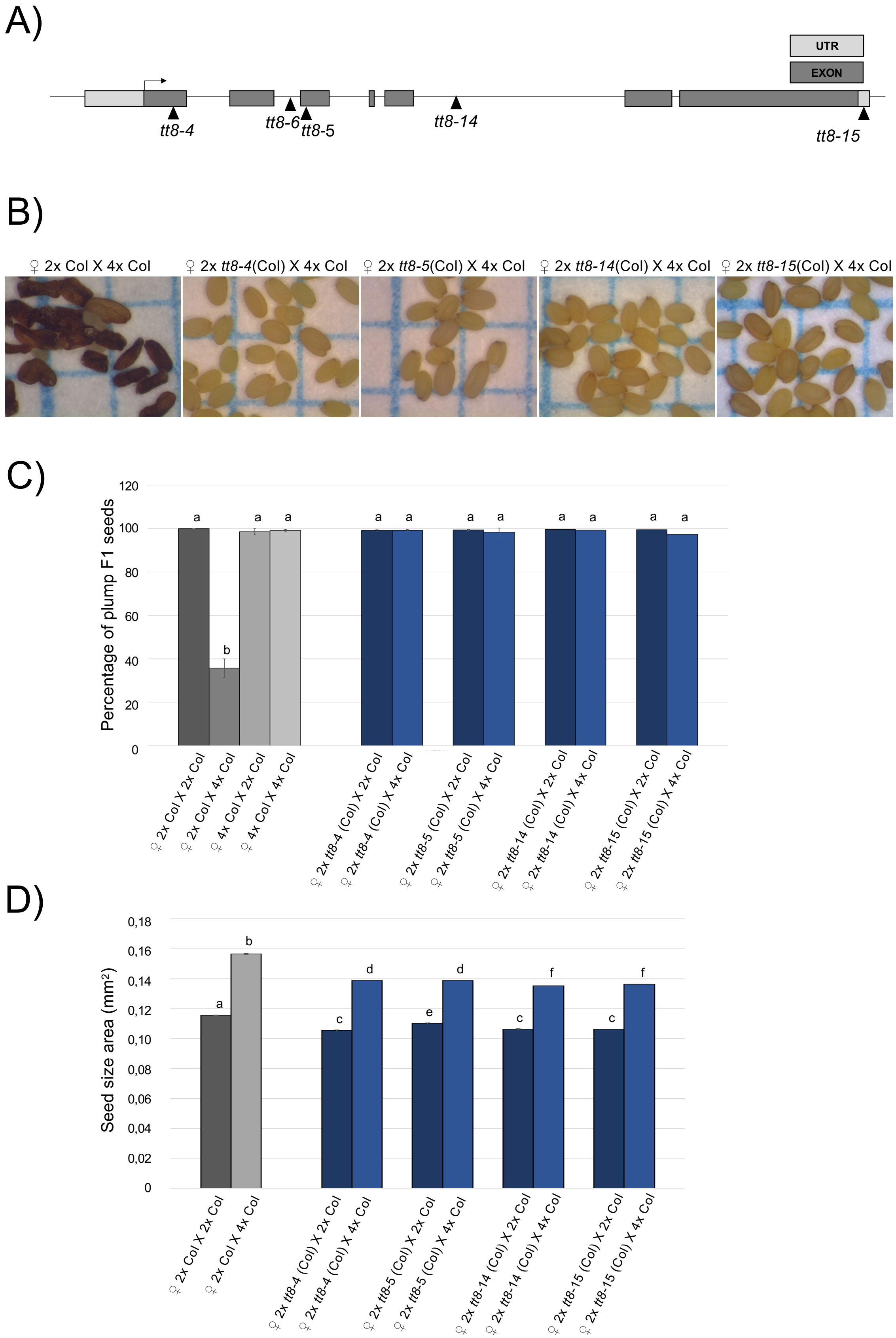

Supplementary Fig. S1. The triploid block "lethal effect" of Columbia interploidy crosses is bypassed by mutation in *TT8.* (a) Localization of *tt8* mutant alleles respect TT8 Genomic locus. (b) Mature F1 seeds obtained crossing different *tt8* mutant alleles with paternal excess Col pollen. (c-d) Percentage of plump F1 seeds c and seed size area d of F1 seeds obtained crossing different *tt8* mutant alleles in paternal-excess crosses. Data are presented as means ± standard error. 200-300 F1 seeds were analysed for each cross. Pooled data of three independent assays performed with 3 replicates for each measurement. One-way ANOVA followed by Tukey HSD test was used for analyzing significance. Crosses marked with the same lowercase letter display no statistical difference.

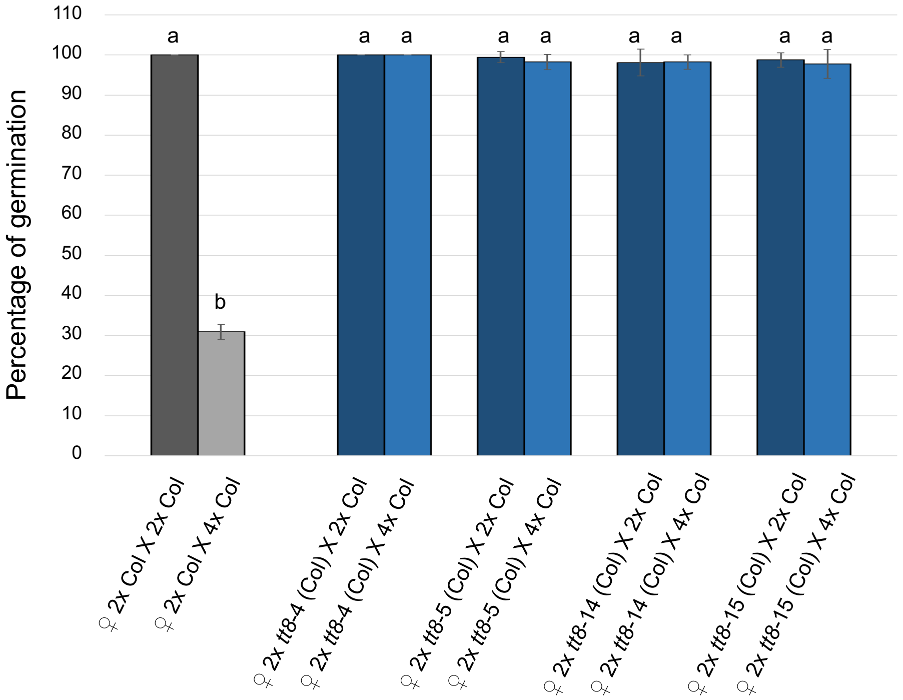

Supplementary Fig. S2. The triploid block impact in germination of Columbia interploidy crosses is bypassed by mutation in *TT8*. Percentage of germinating F1 seeds obtained crossing different *tt8* mutant alleles in paternal-excess crosses. Data are presented as means ± standard error. Pooled data of three independent assays performed with 3 replicates for each measurement. One-way ANOVA followed by Tukey HSD test was used for analysing significance. Crosses marked with the same lowercase letter display no statistical difference.

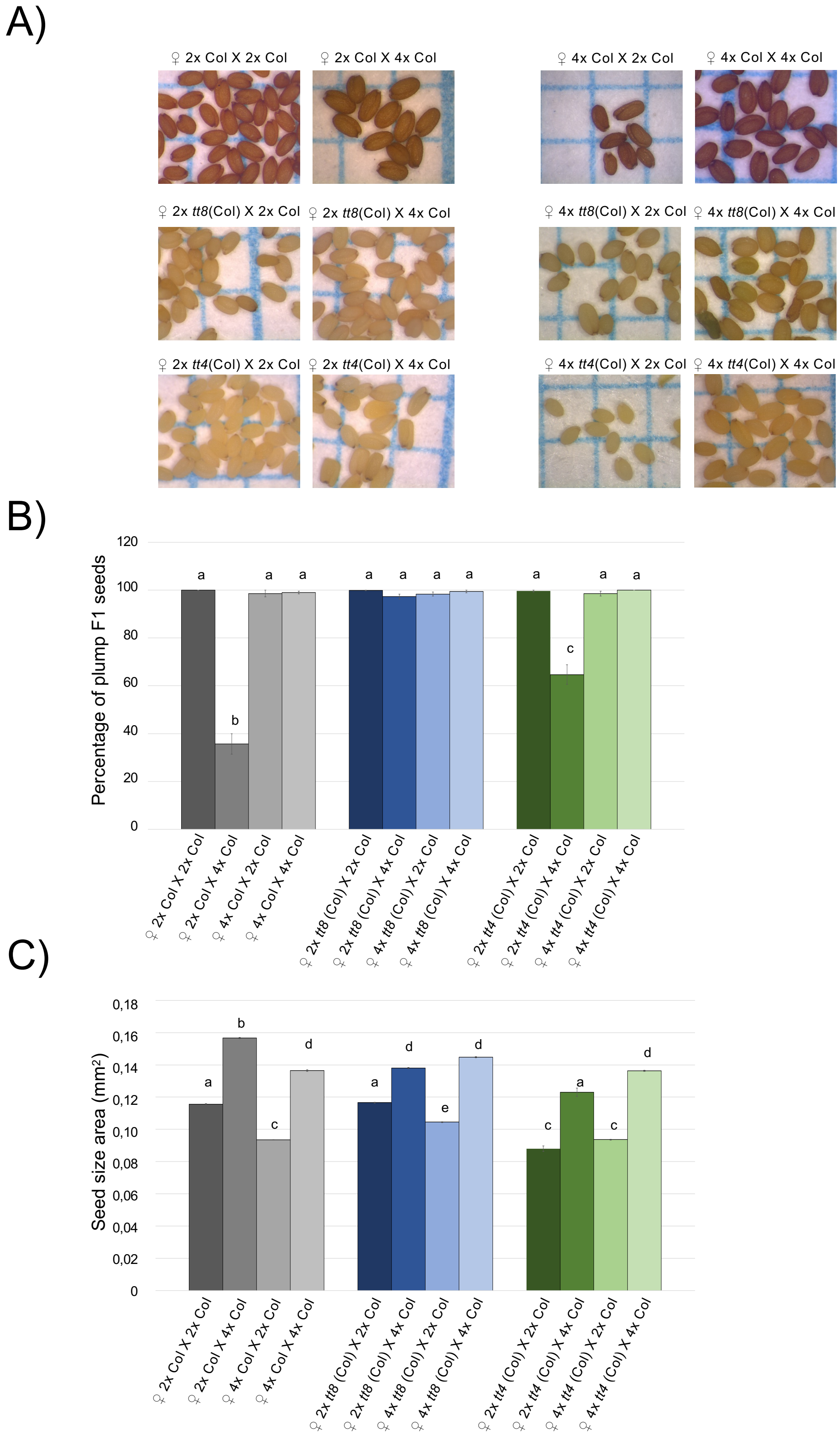

Supplementary Fig. S3. Effects on F1 seed size of *tt8* and *tt4* mutant used in paternal- and maternal-excess interploidy crosses. (a) Mature plump F1 seeds obtained crossing *tt8* and *tt4* mutant pistils in paternal- and maternal-excess crosses. (b-c) Percentage of plump F1 seeds (b) and seed size area (c) of F1 seeds obtained crossing *tt8* and *tt4* mutant pistils in paternal- and maternal-excess crosses. Data are presented as means ± standard error. 200-300 F1 seeds were analyzed for each cross. Pooled data of three independent assays performed with 3 replicates for each measurement. One-way ANOVA followed by Tukey HSD test was used for analysing significance. *tt8* corresponds to *tt8-6 and tt4* is *tt4-11* .Crosses marked with the same lowercase letter display no statistical difference.

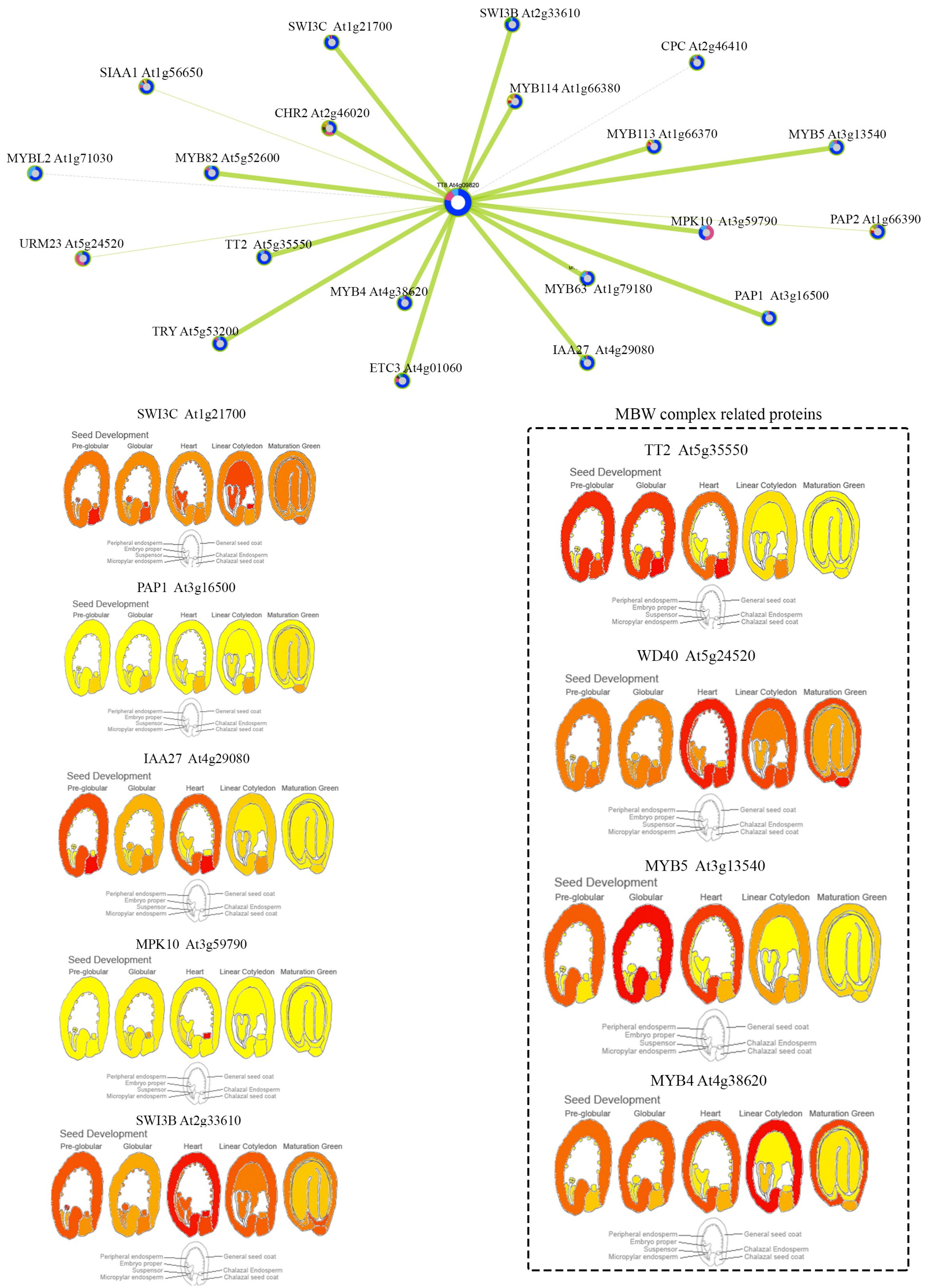

Supplementary Fig. S4. TT8 interactors and their expression in the seed. Data was collected from the interactor viewer 2.0 (http://bar.utoronto.ca/interactions2/) and from the seed eFPbrowswer (http://bar.utoronto.ca/efp_seedcoat/cgi-bin/efpWeb.cgi)

Gene	Primer Sequence
ACT LP	CGTTTCGCTTTCCTTAGTGTTAGCT
ACT RP	AGCGAACGGATCTAGAGACTCACCTTG
TT8 LP	ATGAAGAAGCCGAAGACGAA
TT8 RP	CTTGTGGGGTGTGACATGAG

Supplementary Table S1. Primers used in this study
